# An Unrecognized Fracture of the Neck of the Femur

**Published:** 1879-10

**Authors:** Charles C. F. Gay


### AN UNRECOGNIZED FRACTURE OF THE NECK OF THE FEMUR,

OF NEARLY THREE MONTHS' DURATION—RELIEF OF ALL DEFORMITY BY REFRACTURE—A CLINICAL LECTURE DELIVERED AT THE BUFFALO GENERAL HOSPITAL, BY CHAS. C. F. GAY, M. D.

*Gentlemen*—On December 29, 1877, or more than one year ago, this girl, K. S., then eighteen years of age, was at work in a laundry, and stood at the table ironing nearly all day long; at the close of the day she suddenly became disabled; suffered great pain of the hip and knee, and was put to bed. In about a week she was taken home; on January 19th removed to the Hospital, and on March 14th came under my charge, at which time I noted her right foot was everted, the limb two inches short, and the trochanter two inches further from the anterior superior spinous process of the ilium than that of the opposite side; this side measuring seven and the sound side five inches. There were, at least, two prominent signs, viz.: shortening and eversion of fracture of the neck of the thigh bone; whether it were intra- or extra-capsular or mixed fracture could not be ascertained, and was of little consequence practically, since treatment is the same in either case. The patient stated her limb took this position (everted) from the first, and she let it remain so because it was easier in that position. As regards her previous history, she states she never knew any difference in her limbs, but "one was as good as the other," and she has always been, as at present, strong and healthy.

On March 23d I manipulated the limb, under ether, in the presence of several medical gentlemen and students, in the same or similar manner—except the employment of less force—that I would manipulate a limb in the reduction of a dorsal dislocation by the flexion method, which resulted in relief of all deformity, that is to say: eversion, shortening to the extent of one inch and a half, and the unequal distance between the trochanters and iliac spines were all overcome. Pain, which had been so intense and persistent as to require the use of anodynes, from this time ceased.

Extension, with only eight pounds weight, with the foot of the bed raised two inches, was employed. The day following the manipulations she was quite ill; her pulse ran up to 150 per minute, and it looked as if she might die before the day closed; in which event I should have been held solely responsible for damages. Her alarming condition was attributed, however, to the ether, which was so poorly borne, that, finding her pulse rapidly going up, its administration was not pushed to complete anaesthesia. Her sufferings, aside from that caused by ether, were slight. There was, as a matter of course, some swelling and pain of the thigh, which subsided in two or three days upon the application of warm fomentations. In four weeks extension was discontinued and the girl was allowed to get up and go about upon crutches; she was not able, however, to use them until one or two weeks afterward.

This, in brief, is an outline of the case (a report in detail of which is published in the *Medical Record*, New York, Oct. 12, 1878). To show the results of treatment this girl has kindly consented to come here to-day. I have regarded the case as one possessed of so much surgical interest as to warrant me in inviting this large number present to-day, of the profession of the city, in order that the result may be seen and known, and to give as many as choose an opportunity of measuring the limb, and also that you may be able to see how well the patient can walk without assistance of crutch or cane, the use of which she abandoned a few weeks since.

The heel of the right shoe has been raised half an inch. The girl is able to walk, as you see, with scarcely more halting than has been acquired from habit, and upon careful admeasurement there is not more than half an inch shortening shown. The limb is readily inverted and everted, and the motion of the hip-joint is free and perfect in all respects.

Here, then, is a girl eighteen years of age, suffering intense and protracted pain, having a limb two inches short, and everted with trochanters unequally distant, by two inches, from the iliac spines, and yet, according to her own statement, has received no injury, and there is no history of injury. Her statements may be regarded as reliable; she appears truthful, and there is no possible motive for concealment or suppression of the truth.

Add, now, to the foregoing the additional facts, viz.: that these signs of hip lesion remained unrecognized for nearly three months, and at the expiration of that time manipulations overcame all deformity, and restored the limb to usefulness, and then you will readily come to believe with me, that the case is one of great interest to surgeons, and indeed as remarkable as any hitherto recorded in the history of hip-joint lesions. Sir Astley Cooper affirmed that in case of dislocation of the hip, two months was the time fixed, beyond which it would be imprudent for the surgeon to attempt reduction. But I am not aware that Sir Astley, or any other surgeon, fixed any limitation of time beyond which it would be imprudent to attempt reduction of a badly treated or neglected fracture of the neck of the femur. The result of this case may assist to fix the time when operative measures are to be proscribed.

As the case turned out well, with a result as good as could be desired, and since disability and deformity, which would have been permanent had the limb been " let alone," have been obviated and remedied, no person will deny that the manipulations were proper and wise, and that the end justified the means employed, and furthermore, I think it must be conceded that the case furnishes warrant, if not precedent, for the management of other and similar cases.

Now, what was the condition of this girl's hip that caused the limb to be two inches short, everted, and threw the trochanter two inches away from its normal position? There certainly must have been either fracture or dislocation, but there is only one luxation, and that is the everted dorsal (Bigelow) which involves rupture of the **Y** ligament, that could in any wise be mistaken for it. But the distance of the trochanter from the spine of the ilium would exclude this dislocation. Hence, here is a fracture which, at the expiration of three months, was either united by bone or a long ligament. I suppose it to be almost or quite impossible, by the force ordinarily applied in the reduction of a dorsal dislocation, to fracture the neck of the femur, from the fact that we cannot as firmly hold the upper portion as we can when refracturing the shaft; but a refracture requires less force, and it is quite possible, as in the case before us, to break up a callus, or if you please, refracture the neck of the thigh bone at three or more months after injury.

In answer to the question, how this bone could have been fractured, I reply that fracture of the cervix-femoris does not necessitate great violence or force. Fracture ought not to be overlooked in the absence of violence as a cause. Slight and trivial causes produce fracture, such, for instance, as muscular contraction, a mis-step, a trip upon the carpet, etc. Sir Astley Cooper cites the case of a woman who, "being at her counter, suddenly turning to a drawer behind her, some projection in the floor caught her foot, and preventing its turning with the body, the neck of the thigh bone became fractured."

At the age of this patient, separation at the epiphysis would be likely to occur with less degree of violence than would be required to produce a fracture at the diaphysis. The slight amount of shortening is indicative of non-absorption of the neck of the femur, and is suggestive of epiphysial fracture. I must not omit to call attention especially to the fact that, although the existence of fracture was established beyond scarcely any reasonable doubt, yet, during the final manipulations, a jerking impulse was conveyed to the hand, and a sound heard such as we are prepared to hear whenever a dislocation is reduced, which was so audible as to be heard by all, that gave rise to a mooted question whether or not it were a fracture or dislocation. Indeed, the resemblance was so close as to deceive some of those who were present into the belief that a coxo-femoral dislocation had been reduced. In order to resolve the question of doubt, the manoeuvre of flexion, rotation, abduction and extension was twice repeated, with the effect of eliciting the same audible snap, showing conclusively that it was caused by re-adjustment and coaptation of the ends of broken bones.

After reduction, and during the time of making extension, the upper and lower fragments were held in apposition by three or four bands of adhesive plaster, two inches wide, and long enough to embrace the circumference of the pelvis and include the trochanters. These bands also served to prevent eversion, and it is the best appliance for this purpose in the treatment of intra- or extra-capsular fracture.

I am quite fully persuaded that osseous union is possible in intra-capsular fracture. Non-union is usually attributed by writers—including Sir Astley Cooper— to want of nourishment of the detached head of the femur; but in all the post-mortem specimens I have examined, there has been no such evidence; indeed, the femoral heads of specimens I have examined have been well nourished, and have retained their normal size and symmetry. I apprehend the chief cause of non-union to be, non-apposition of the ends of the broken bones, and, as Sayre puts it, want of fixation after apposition.

